# Public health round-up

**DOI:** 10.2471/BLT.17.010517

**Published:** 2017-05-01

**Campaign highlights the need for full immunization**

Children hold up their vaccination cards during the 2016 yellow fever outbreak in Angola. This year’s World Immunization Week campaign, from 24–30 April, aims to raise awareness about the importance of full immunization throughout life, and its role in achieving the 2030 sustainable development goals.

http://www.who.int/campaigns/immunization-week/2017/

**Figure:**
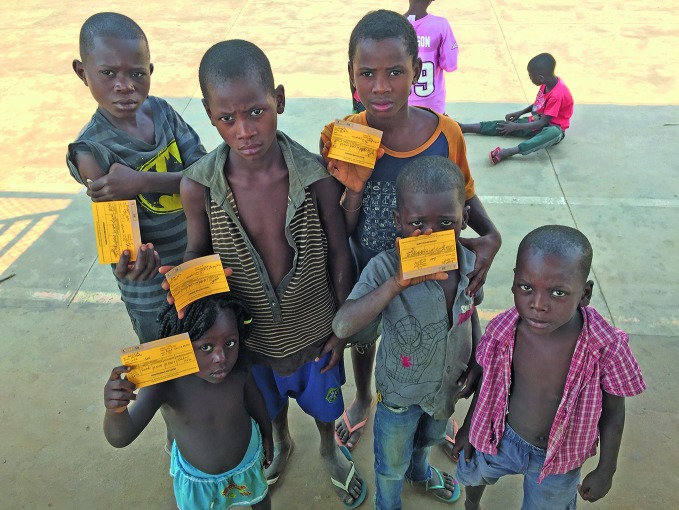
WHO /Dalia Lourenço

## WHO alarmed by chemical weapons reports 

The head of the World Health Organization’s (WHO) Health Emergencies Programme expressed alarm at reports of the use of highly toxic chemicals in an attack in the Syrian Arab Republic on 5 April. 

At least 70 people died and hundreds more were affected in Khan Shaykhun in the governorate of Idleb, according to United Nations Health Cluster partners who were treating patients – many of them women and children – with breathing difficulties.

“The images and reports coming from Idleb today leave me shocked, saddened and outraged. These types of weapons are banned by international law because they represent an intolerable barbarism,” said Dr Peter Salama, Executive Director of the WHO Health Emergencies Programme.

Since 2012, when the first reports emerged of the use of chemicals as weapons in the Syrian conflict, WHO has been engaged in public health preparedness for the management of patients exposed to chemical or toxic gas. 

This included issuing new clinical management protocols, preparing hospitals to receive and treat patients, distributing protective equipment to hospitals, and raising awareness amongst Syrians on how they can protect themselves against exposure and when to seek treatment. 

In 2016, WHO trained 200 clinicians on the initial management of chemical weapons cases including pre-hospital decontamination, referral, triage and treatment. 

An additional 65 doctors in northern Syrian were trained by WHO’s field office in Gaziantep, southern Turkey. The majority of doctors trained were from Idleb, the governorate where the 5 April attack took place. 

http://www.who.int/mediacentre/news/statements/2017/toxic-chemicals-syria/

## Aiming for zero malaria cases

The World Health Organization released new policy guidance last month to aid programme managers in their development of national strategic plans for the elimination of malaria.

A framework for malaria elimination provides countries with a set of tools and strategies for achieving and maintaining elimination, regardless of where they lie across the continuum of transmission.

Malaria is a potentially fatal disease caused by parasites transmitted to people through the bites of infected female *Anopheles* mosquitoes. In 2015, 91 countries and areas had ongoing malaria transmission.

The framework, which supersedes the 2007 malaria elimination field manual, was developed through a broad, consultative process led by the WHO Secretariat and an independent 13-member evidence review group of international experts from diverse fields.

Several countries have recently eliminated the disease, including Kyrgyzstan, the Maldives and Sri Lanka.

In addition, 21 countries are on track to reduce malaria transmission to zero by 2020 and are being supported by WHO to achieve this target.

WHO certification of malaria elimination requires that local transmission of all human malaria parasites has been interrupted nationwide, resulting in zero incidence of indigenous cases for at least three consecutive years. The certification process has been streamlined, and is described in the new framework.

In addition, the concept of subnational verification of malaria elimination has been introduced in the new malaria guidance and is an option for large countries that have achieved interruption of local transmission in certain parts of the country.

Subnational verification can be an important building block for future national certification.

http://www.who.int/malaria/publications/atoz/9789241511988

## WHO ethics guidance for TB

New tuberculosis ethics guidance was launched ahead of World TB Day on 24 March by the World Health Organization.

The guidance can be used by governments, health workers, care providers, nongovernmental organizations, researchers and others implementing the End TB Strategy.

The aim is to make all tuberculosis stakeholders aware of their responsibilities, in terms of adhering to sound ethical standards and protecting the rights of those affected.

Protecting human rights, ethics and equity are principles which underpin WHO’s End TB Strategy. But it is not easy to apply these principles on the ground. Patients, communities, health workers, policy-makers and other stakeholders frequently face conflicts and ethical dilemmas. The current multidrug-resistant TB (MDR-TB) crisis and the health security threat it poses accentuate the situation even further.

Poverty, malnutrition, poor housing and sanitation, compounded by other risk factors such as HIV, tobacco, alcohol use and diabetes, can put people at heightened risk of tuberculosis and make it harder for them to access care.

The new WHO ethics guidance addresses contentious issues including the isolation of contagious patients, the rights of tuberculosis patients in prison and discriminatory policies against migrants affected by tuberculosis.

It emphasizes five key ethical obligations to: provide patients with social support; to refrain from isolating tuberculosis patients before exhausting all options to enable treatment adherence and only under very specific conditions; enable key populations to access the same standard of care offered to other citizens; ensure that all health workers operate in a safe environment; rapidly share evidence from research to inform national and global tuberculosis policy updates.

http://www.who.int/mediacentre/news/releases/2017/world-tb-day

## Health care in South Sudan

South Sudan launched a new community health initiative last month. Currently, only 44% of people in this sub-Saharan country of some 12 million people are within reach of health facilities.

The Boma Health Initiative – named after the smallest administrative unit in the country – aims to provide sustainable delivery of essential health care and public health programmes at the community level.

Under the initiative, three people from each boma will be trained as community health workers and equipped to deliver high impact, cost-effective primary health-care services.

Initially, the Boma Health Service Package will cover maternal and child care, as well as HIV, malaria, tuberculosis and some neglected tropical diseases.

The community health workers will also be expected to report births and deaths including maternal deaths, and will be responsible at the boma level for integrated disease surveillance.

They will also gather vital statistics for the national health management information system.

The initiative is being rolled out by the ministry of health with support from WHO and other partners.

Communicable diseases are a leading cause of mortality and morbidity in South Sudan, and the population is at risk of disease outbreaks.

Malaria is one of the biggest causes of illness and death, while cholera, measles and kala-azar are also major health threats.

http://www.afro.who.int/en/ssd/news.html

**Cover photo**

This worker is manufacturing medical equipment in a grinding mill in Sialkot, Pakistan. Such workers work long hours for as little as US$ 2 a day. In addition, they may be exposed to occupational hazards, including risks from poor electrical wiring, and toxic and corrosive chemicals.

**Figure:**
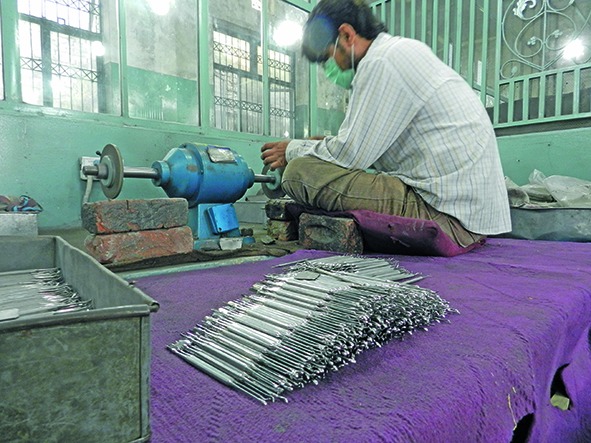
British Medical Association/Arthy Santhakumar

## Medication without harm

WHO launched a new global initiative on 29 March to reduce unsafe medication practices and medication errors, a leading cause of injury and health care-associated harm globally.

The goal is to reduce the level of severe avoidable harm related to medications by 50% over five years globally. Medication-related harm will be measured in terms of medication-related hospital admissions, readmissions and deaths. 

A WHO Working Group on Monitoring and Evaluation has been set up under the Third WHO Global Patient Safety Challenge on medication safety, comprising international multidisciplinary experts in patient safety to track progress towards this goal. 

The challenge primarily aims to improve ordering, prescribing, dispensing, administering and monitoring of medications. WHO will provide guidance and develop strategies, plans and tools that countries can use to make the medication process safer. 

This is WHO’s third global patient safety challenge, following the Clean Care is Safe Care challenge on hand hygiene in 2005 and the Safe Surgery Saves Lives challenge in 2008.

http://who.int/mediacentre/news/releases/2017/medication-related-errors

## Reporting on health systems

A portrait of the health system in the world’s fourth most populous country, Indonesia, was released last month by the Asia Pacific Observatory on Health Systems.

The Health in Transition (HiT) report on Indonesia highlights recent efforts to increase health-care coverage with the introduction of the National Health Insurance Scheme in 2014.

The goal is to achieve universal health coverage by 2019 in this country of some 257 million people spread across about 900 permanently inhabited islands with significant devolution of authority to provinces and districts.

Other reports in the series are on: Bangladesh, Cambodia, China, Fiji, Lao People’s Democratic Republic, New Zealand, Malaysia, Mongolia, Myanmar, Philippines, Republic of Korea, Solomon Islands, Thailand and Tonga.

http://www.wpro.who.int/asia_pacific_observatory/hits/latest_hits

**Looking ahead**

10–12 May – High-Level eHealth Conference, Malta. This year’s theme is “data for health: the key to personalized sustainable care”

22–31 May – 70th World Health Assembly, Geneva, Switzerland

31 May – World No Tobacco Day. This year’s theme is “tobacco: a threat to development”

